# Morphosyntactic development in German-speaking individuals with Down syndrome—longitudinal data

**DOI:** 10.3389/fpsyg.2023.1118659

**Published:** 2023-06-21

**Authors:** Bernadette Witecy, Eva Wimmer, Isabel Neitzel, Martina Penke

**Affiliations:** ^1^Department of Rehabilitation and Special Education, University of Cologne, Cologne, Germany; ^2^Department of Rehabilitation Sciences, TU Dortmund University, Dortmund, Germany

**Keywords:** Down syndrome, language development, expressive grammar, receptive grammar, subject-verb agreement, *wh*-questions, verbal short-term memory, longitudinal study

## Abstract

**Introduction:**

The present study provides longitudinal data on the development of receptive and expressive grammar in children and adolescents with Down syndrome and addresses the role of nonverbal cognitive abilities and verbal short-term memory for morphosyntactic development.

**Method:**

Seventeen German-speaking individuals with Down syndrome (aged 4;6–17;1 years at first testing (T1)) were assessed twice, 4;4–6;6 years apart. For a subset of five participants, there was also a third assessment 2 years after the second. Receptive grammar, nonverbal cognition, and verbal short-term memory were tested using standardized measures. For expressive grammar, elicitation tasks were used to assess the production of subject-verb agreement and of *wh*-questions.

**Results:**

At group level, the participants showed a significant increase in grammar comprehension from T1 to T2. However, progress diminished with increasing chronological age. Notable growth could not be observed beyond the age of 10 years.

With respect to expressive grammatical abilities, progress was limited to those participants who had mastered verbal agreement inflection around age 10 years. Individuals who did not master verbal agreement by late childhood achieved no progress in producing *wh*-questions, either.

There was an increase in nonverbal cognitive abilities in the majority of participants. Results for verbal short-term memory followed a similar pattern as those for grammar comprehension. Finally, neither nonverbal cognition nor verbal short-term memory were related to changes in receptive or expressive grammar.

**Discussion:**

The results point to a slowdown in the acquisition of receptive grammar which starts before the teenage years. For expressive grammar, improvement in *wh-*question production only occurred in individuals with good performance in subject-verb agreement marking, which suggests that the latter might have a trigger function for further grammatical development in German-speaking individuals with Down syndrome. The study provides no indication that nonverbal cognitive abilities or verbal short-term memory performance determined the receptive or expressive development. The results lead to clinical implications for language therapy.

## 1. Introduction

Down syndrome is one of the most common genetic disorders, caused by a third copy of chromosome 21 or part thereof (Martin et al., 2009; Loane et al., 2013). It is associated with both intellectual disability and language deficits. Language acquisition is overall delayed, but not all language domains are affected to the same extent. Individuals with Down syndrome often display severe impairments in the area of morphosyntax (for overview see Abbeduto et al., 2007; Roberts et al., 2008) as opposed to vocabulary skills or communicative and pragmatic competencies (Vicari et al., 2000; Grieco et al., 2015).

Receptive grammatical abilities are often considered to be less affected than expressive skills (Chapman et al., 1998), but many individuals with Down syndrome still exhibit difficulties in sentence comprehension (see review by Andreou and Chartomatsidou, 2020). Such difficulties often limit the comprehension of so-called non-canonical sentences in which the word order does not correspond to the unmarked constituent structure in a given language (e.g., passives or object-initial questions; Wimmer and Penke, 2020). They can, however, also affect the comprehension of syntactically simple sentences, such as simple active sentences (Witecy and Penke, 2017).

Sentence production can be limited to short utterances (Fowler et al., 1994) and is often characterized by frequent omissions of free and bound grammatical morphemes (Chapman et al., 1998). If longer utterances are produced, they can be incoherent and fragmental (Neitzel and Penke, 2021). Studies that used elicitation techniques to assess specific sentence structures revealed considerable difficulties with the production of syntactically complex sentences, for example different kinds of *wh*-questions (Tsakiridou, 2006; Joffe and Varlokosta, 2007; Wimmer et al., 2020). Studies analyzing spontaneous speech or data from elicitation or sentence repetition tasks also observed a particular deficit with the production of inflectional morphology in individuals with Down syndrome (e.g., Eadie et al., 2002; Laws and Bishop, 2003; Caselli et al., 2008; Penke, 2018). Most prominent are difficulties with verbal agreement and/or tense inflection that were found for different languages, such as English (e.g., Eadie et al., 2002; Laws and Bishop, 2003), Dutch (Bol and Kuiken, 1990), or German (Penke, 2018). In language production such affixes are often omitted or substituted by markers expressing different grammatical information (for instance, nonfinite markers; Penke, 2018).

Despite the existence of overarching symptoms that characterize the language difficulties in Down syndrome, individuals with Down syndrome exhibit large individual variability with respect to their language abilities. In many cases grammatical comprehension and production are impaired as described above, and some individuals with Down syndrome continue to show difficulties even with basic morphosyntactic structures into adolescence or adulthood (Fowler et al., 1994; Rondal and Comblain, 1996). Others, however, are able to comprehend or produce complex sentences (e.g., Thordardottir et al., 2002), and some individuals even appear to have nearly unimpaired language abilities (e.g., Rondal, 1995).

Besides the individual variability in language achievements in individuals with Down syndrome, a common finding is that the morphosyntactic development of individuals with Down syndrome is protracted and lags considerably behind chronological and often also mental age (e.g., Chapman et al., 1998). This raises questions regarding the timeframe in which morphosyntactic abilities are likely to develop further. In a cross-sectional study with 58 participants with Down syndrome, aged 4;6–40;3 years; Witecy and Penke (2017) found a positive correlation of grammar comprehension abilities with chronological age in children and adolescents (up to the age of 20 years), but not in adults. Similar results were reported by Facon and Magis (2019) and Iacono et al. (2010). Correlational analyses in these studies revealed a positive relation between chronological age and language comprehension measured through standardized tests in a group of children and adolescents (*n* = 62, aged 7–22 years; Facon and Magis, 2019), but not in adults (*n* = 55, aged 19–58 years; Iacono et al., 2010). Taken together, these findings suggest an ongoing development of receptive grammar in adolescence and subsequently, the building of a plateau in adulthood.

Rondal and Comblain (1996) conclude from their own and other cross-sectional data that grammatical development already ends around the age of 12–14 years in individuals with Down syndrome, not only in the receptive but also in the expressive domain. In contrast, based on the finding that the mean length of utterance (MLU) in narrative discourse increased with chronological age in a cross-sectional sample of 24 participants with Down syndrome (12;5–20;4 years), Thordardottir et al. (2002) assert that expressive syntactic development proceeds into late adolescence. Iacono et al. (2010)—in contrast—found a negative correlation between age and utterance lengths in narratives in their sample of adult participants.

Although these cross-sectional studies give some indications regarding the development of grammar, longitudinal data are needed to draw reliable conclusions on developmental trajectories in individuals with Down syndrome. The number of studies that have examined the development of grammatical abilities in individuals with Down syndrome longitudinally is, however, scarce, especially for expressive grammar. With respect to grammar comprehension, Conners et al. (2018) did not find significant growth (receptive grammar measured by the *Test for Reception of Grammar*, 2nd edition (TROG-2); Bishop, 2003) in a sample of 42 individuals with Down syndrome (chronological age 10–21 years at Time 1) over a period of 2 years. It is possible though, that progress in individuals with Down syndrome is slow and could therefore not be detected due to the relatively short time span that elapsed between the first and the second measurement (2 years later) in this study. Indeed, in a study by Laws and Gunn (2004) with two assessments 5 years apart, increases in grammar comprehension (measured by TROG scores) could be observed in most of the 30 participants (chronological age at study start: 5–19 years). However, younger participants made more progress than older ones and some of the latter even showed declining scores. Chapman et al. (2002) estimated individual growth functions for syntax comprehension (measured by the *Test of Auditory Comprehension of Language–Revised* (TACL-R), Carrow-Woolfolk, 1985) using hierarchical linear modelling. They collected data of 31 participants with Down syndrome who were tested four times over a period of 6 years (chronological age at first assessment: 5–20 years). The model predicted that there is still some progress in comprehension abilities in individuals aged around 12 years, but that the receptive skills are likely to decline in individuals aged 17 years or older, that is, in late adolescence and early adulthood.

Regarding expressive grammar, existing longitudinal evidence is conflicting as to whether there is improvement with increasing chronological age in adolescence or not. Support for the former comes from the study by Chapman et al. (2002) who estimated individual growth functions not only for receptive but also for productive syntactic abilities. In the four assessments over the span of 6 years, they found ongoing development in language production as measured by MLU, obtained in narrative samples, for most of the participants. Growth in expressive syntax over 2 years was also reported by Martin et al. (2013). They assessed expressive syntactic abilities using a standardized measure (Syntax Construction subtest of *Comprehensive Assessment of Spoken Language, CASL*; Carrow-Woolfolk, 1999) in up to three waves, each 1 year apart, in a varying number of boys with Down syndrome (Time 1: *n* = 32, Time 2: *n* = 21, Time 3: *n* = 16). Conners et al. (2018), on the other hand, did not observe progress in grammar production, neither in a standardized measure of expressive morphology (the word structure subtest of the CELF-P2; Wiig et al., 2004) nor in MLU based on narrations elicited by wordless picture books, a finding which conforms to their observation regarding receptive grammatical abilities. Again, as already mentioned above and as the authors state themselves, growth might have been so modest that it did not become apparent in the time span of 2 years elapsing between first and second testing of the participants in this study.

In summary, previous investigations point to a slowdown of the development in receptive grammar in adolescence which is followed by a plateau or even a decline. It remains unclear, however, whether the same applies to productive grammatical abilities or whether there is ongoing growth in adolescence. Furthermore, the question arises, which factors play a role in determining the course of development.

One factor that has been discussed to be associated with the language development in individuals with Down syndrome are nonverbal cognitive abilities or rather the limitations in this respect. Nonverbal cognitive abilities include visual–spatial processing and inductive reasoning skills. They are usually measured using standardized tests or subtests thereof that require only minimal or no verbal instructions (e.g., Leiter-R (Roid and Miller, 1997), Stanford-Binet 4th edition (Thorndike et al., 1986), Raven’s Colored Progressive Matrices (Raven et al., 1995), Snijders-Omen Nonverbal Intelligence Test (Tellegen et al., 2007)). A number of cross-sectional studies have revealed a positive relationship between receptive or expressive grammatical abilities and nonverbal cognition in individuals with Down syndrome (Chapman et al., 1991; Abbeduto et al., 2003; Aktaş, 2004; Price et al., 2007, 2008; Iacono et al., 2010; Estigarribia et al., 2012; Finestack et al., 2013). That is, higher nonverbal cognitive abilities, assessed using the aforementioned measures, were correlated with higher grammatical abilities. Due to the fact that only one point in time is measured in these investigations, it is, however, unclear whether the observed relation indeed reflects a developmental association. Regarding the development of nonverbal cognitive abilities themselves, previous research has reported slowed, but continuing growth into adulthood (Couzens et al., 2011; Channell et al., 2014; Grieco et al., 2015). This ongoing development contrasts with the just presented studies on the development of grammatical abilities that have observed a standstill in receptive and/or expressive grammar (Chapman et al., 2002; Laws and Gunn, 2004; Conners et al., 2018). The question is whether this speaks against nonverbal cognition as a determining factor for the development of grammar or whether variation in nonverbal cognitive abilities still might be a predictor. First longitudinal evidence for the former view is provided by Chapman et al. (2002). They did not find nonverbal cognition, as measured by the Pattern Analysis subtest from the Stanford-Binet 4th edition, which assesses visual–spatial processing (Thorndike et al., 1986), to predict individual growth in grammar comprehension or production. In Conners et al. (2018) and Martin et al. (2013) nonverbal cognitive ability was included as a covariate in data analysis, but its role as a predictor for grammar development was not explicitly analyzed. We are not aware of any other longitudinal studies that have examined the relation between nonverbal cognitive abilities and grammar development to this date. Thus, further research in this respect is needed.

Apart from nonverbal cognition, morphosyntactic development could be influenced by weak verbal short-term memory skills that constitute another characteristic symptom in individuals with Down syndrome (*cf.* meta-analyses by Næss et al., 2011; Godfrey and Lee, 2018). According to Baddeley’s influential multicomponent model on working memory (e.g., Baddeley, 2003; Baddeley et al., 2021), verbal short-term memory represents the memory component most relevant for language. It comprises a passive capacity-restricted phonological store that maintains phonological information (e.g., words and sentences) for up to 2 s. Memory traces can be refreshed by a rehearsal process, a kind of inner speech. The crucial role of this phonological loop component consisting of storage and rehearsal is to enable the hearer to extract the relevant morphosyntactic information from the speech signal during processing, a prerequisite to language comprehension and grammar development. Verbal short-term memory skills have been shown to play an important role in typical and atypical acquisition of morphosyntax (*cf.* recent papers by Delage and Frauenfelder, 2020; Roesch and Chondrogianni, 2021).

In most studies on Down syndrome that relate morphosyntactic comprehension or production skills to the performance in verbal short-term memory tasks, significant relations between the two domains have been found (e.g., Laws and Bishop, 2003; Laws and Gunn, 2004; Miolo et al., 2005; Estigarribia et al., 2012; Frizelle et al., 2019b; Wimmer et al., 2020). However, the majority of these studies are cross-sectional and the observed relations might also be due to task demands. This might especially hold for grammar comprehension which is usually assessed using sentence picture-matching tasks that place high demands on verbal short-term memory (*cf.* Frizelle et al., 2019a; Penke and Wimmer, 2020 for discussion). Longitudinal studies are rare so far, but existing studies have found verbal short-term memory capacity—measured by nonword repetition—to be a predictor for progress in grammar comprehension (Chapman et al., 2002; Laws and Gunn, 2004). However, in the investigation by Laws and Gunn (2004), this only held for the younger participants that were aged below 10 years at initial assessment. This suggests that verbal short-term memory may play an important role for the acquisition of receptive grammar, especially in childhood and early adolescence, but that it might be less relevant for progress of language abilities in older individuals with Down syndrome.

Whether verbal short-term memory capacities are also associated with the development of expressive morphosyntactic abilities in individuals with Down syndrome, is unclear yet. In the study by Chapman et al. (2002), performance with respect to verbal short-term memory did not predict development in expressive grammar (as measured by MLU). However, growth in expressive grammar was predicted by abilities in grammar comprehension which in turn were related to short-term memory capacity. Hence, there might be an indirect relation between the latter and productive grammatical abilities mediated by comprehension abilities.

To summarize, the following open issues arise from the current state of research: Whereas existing studies reveal a clear tendency for the developmental course in grammar comprehension, namely, a levelling off of the development in adolescence, the picture is less clear for expressive morphosyntactic abilities. Furthermore, more longitudinal research is needed that targets the role of nonverbal cognition and verbal short-term memory as potential influencing factors for morphosyntactic development in production and comprehension. Against this background, the aim of our study is to investigate both grammar comprehension and production as well as the described potential predictor variables longitudinally in the same sample of individuals with Down syndrome.^1^ In doing so, we will not only look at the performance of the group, but we will focus on individual development. Explicit investigations of individual differences have often been neglected in previous studies on Down syndrome, but seem important given the reports of large inter-subject variability in the literature (Conners et al., 2018).

Difficulties in grammar comprehension or production can negatively affect the communication and participation of individuals with Down syndrome, as they may be less able to follow conversations and prompts in their environment or to express their needs and thoughts. This is particularly true in educational or employment settings. In addition, impaired grammar comprehension can also significantly impede intervention in other language areas, such as vocabulary or expressive morphosyntax. Thus, understanding both the nature of the receptive and expressive grammatical difficulties and their developmental course is important for practitioners in therapeutic as well as educational and vocational contexts.

The research questions of the current investigation are: (RQ1) What is the course of development in (a) receptive and (b) expressive morphosyntactic abilities in individuals with Down syndrome? (RQ2) What is the role of nonverbal cognition and verbal short-term memory in determining the developmental progress in morphosyntax?

## 2. Materials and methods

### 2.1. Participants

Seventeen German-speaking individuals with Down syndrome (7 female, 10 male; chronological age at study start: 4;6–17;1 years) were assessed twice, 4;4–6;6 years apart.^2^ Information on the chronological ages of the participants at the different points of assessment are presented in Table 1 (see Supplementary material for individual data). The nonverbal mental age of the participants ranged between 3;5 and 6;5 years at the initial assessments (T1) (*M =* 4;8, *SD* = 1;0). Participants were included in the study if they were monolingual German speakers, used oral language as their primary means of communication, and produced at least two-word-utterances. This was confirmed by the parents, and the latter two aspects were additionally checked during the first assessment session. At T1 participants’ parents were asked to fill out a questionnaire to provide information on biographical and medical background, including questions on hearing and ear infections, kindergarten/school attendance, speech and language therapy, and their own level of education. At the second testing (T2), a further questionnaire was given to follow up on part of these aspects. All children, adolescents, and young adults in the present study had normal or corrected vision as well as normal hearing, with the exception of one participant who was reported to have a mild conductive hearing loss of 35 dB in one ear. The participants all attended inclusive kindergartens or inclusive or specials needs schools at T1 and were still in school at T2, apart from one young adult who was working in a sheltered employment facility at the time of the second assessment. To gain a more comprehensive picture of the development of expressive grammatical abilities, we tested those five individuals of the initial cohort of 17 participants a third time (T3) who had not mastered the grammatical structures under study at T2. This third testing took place 2 years after the second assessment and 8 years after the first (see Table 1).

**Table 1:** Overview of participants (ages in years; months).

*N*	Sex	Chronological age at T1	Chronological age at T2	Time span from T1 to T2
17	7 female 10 male	Range: 4;6–17;1 *M* = 9;10 (*SD =* 3;3) *Mdn* = 9;6	Range: 11;0–23;2 *M* = 15;7 (SD = 3;3) *Mdn* = 15;5	Range: 4;4–6;6 years *M* = 5;9 (*SD* = 0;8) *Mdn* = 6;0
Retested at T3				Chronological age at T3
5	2 female 3 male	Range 4;6–12;0 *M* = 8;8 *Mdn* = 8	Range: 11;0–18;1 *M* = 14;9 *Mdn* = 13;8	Range: 13;0–20;0 *M* = 16;8 *Mdn* = 15;7

### 2.2. Measures

#### 2.2.1. Language measures (RQ1)

##### 2.2.1.1. Receptive grammar: TROG-D

The standardized measure TROG-D (Fox, 2011), a German adaption of the Test for Reception of Grammar (Bishop, 1983, 2003), was applied to assess grammar comprehension. The TROG is widely used in research of language development in different populations and languages as well as in clinical practice. It has also been employed in the longitudinal studies by Laws and Gunn (2004) and Conners et al. (2018) to assess receptive grammatical abilities in individuals with Down syndrome. The internal consistency reliability (Cronbach’s *α*) of the TROG-D is 0.90. The TROG-D is correlated with the sentence comprehension subtest from the SETK 3-5 (Sprachentwicklungstest für drei- bis fünfjährige Kinder “Test of language development for three to five year old children”; Grimm, 2001) at *r* = .72 (Fox, 2011).

Participants were verbally presented with a word or a sentence and had to choose the corresponding picture out of a choice of four. The test includes 21 blocks of four items each. Each block tests a different grammatical structure which increases in grammatical complexity. In accordance with the manual, testing was discontinued when the participant gave at least one incorrect answer in five consecutive blocks. Raw scores were used for the analyses. They result from the number of blocks, in which all items have been answered correctly, and therefore might range between 0 and 21 points.

##### 2.2.1.2. Expressive grammar: elicitation tasks on subject-verb agreement and *wh*-question production

Previous studies on productive grammatical abilities have employed MLU as a measure indicating grammatical complexity. MLU provides an indirect measure of morphosyntactic development, the assumption being that the longer the utterance the more complex the structure. For individuals with Down syndrome, however, this assumption does not seem to hold. In an investigation of narrations produced by German individuals with Down syndrome, we found high MLU values to often come about by ungrammatical concatenations of sentence fragments within one utterance (Neitzel and Penke, 2021). Here, we therefore adopted two elicitation tasks to assess expressive grammatical abilities more directly: one focusing on verbal agreement inflection and the other on *wh-*question production. Both phenomena have been found to often be affected in individuals with Down syndrome (e.g. Penke, 2018; Wimmer et al., 2020) and the two tasks have been applied successfully to assess these phenomena in individuals with language impairments in the past.

A video description task was performed to elicit verb forms marked for subject-verb agreement. Participants had to describe the action depicted in 30 short, silent video scenes presented on a laptop computer. They were prompted by the question *Was passiert da?* “What is happening here?”. In the videos the participants could either see the experimenter herself, a single child, or two children performing an action and were therefore expected to produce verbs inflected for 2nd person singular (e.g., *du schreib-st* “you are writing” for videos showing the experimenter), 3rd person singular (e.g., *er koch-t* “he is cooking” for videos showing a single child), or 3rd person plural (e.g., *sie lauf-en* “they are running” showing two children). First, participants were familiarized with the task by three practice items in which the acting characters were introduced. Subsequently, there were 10 target videos for each grammatical context (2nd person singular, 3rd person singular, 3rd person plural). All 30 target videos were presented in a previously fixed randomized order. Accuracy scores for correct agreement inflection were determined for all utterances that consisted of both an overt subject and a main verb. An utterance was scored as correct if the suffix on the verb agreed with the subject. Both unmarked verbs and substitutions of the ending were considered incorrect.^3^

In addition, we assessed the production of complex syntactic structures by eliciting *wh*-questions. We collapsed data that came from two methodically comparable tasks eliciting *wh*-questions (see Table 2). In both tasks, participants were instructed to pose different *wh-*questions to either a toy figurine or to toy animals (e.g., “Ask the snail what it is doing.”; see Table 2 for more details on the item material). At T1 only Task 1 was used.^4^ At T2 six participants were administered Task 1 and seven were assessed using Task 2. As the structure and content of the questions as well as the method of elicitation in a playful setting and the number of questions are comparable in the two tasks, we combined the data at T2. Similar question elicitation tasks with puppet scenarios or pictures are common and adequate tools to evaluate expressive grammatical abilities in children (*cf.* Thornton, 1996). They have been used successfully in the past in children with developmental language disorders of diverse etiology (for Down syndrome and Williams syndrome, e.g., Joffe and Varlokosta, 2007, for children with Autism Spectrum Disorder and children with Developmental Language Disorder *cf.* Sukenik et al., 2021).

**Table 2:** Overview of task and item material for *wh*-question production tasks 1 and 2.

	*Wh*-questions task 1	*Wh*-questions task 2
*n* items overall	*n* = 14	*n* = 12
instrument	‘Ask the snail’ game (*cf.* Wimmer et al., 2020)	Subtest 1 of ESGRAF 4–8, Item 1–12 (Motsch and Rietz, 2016)
task	Pose questions to a figurine (snail/robot) (structured dialogue)	Pose questions to identify three toy animals (monkey, pig, goose) hidden in a box
instruction	Frag die Schnecke, was sie hier macht. (“Ask the snail what it is doing.”)	Frag das Tier, was es fressen mag. (“Ask the animal what it would like to eat.”)
*wh-*argument questions	*n* = 8 *wh*-subject and-object questions (*who/what* questions)	*n* = 6 *wh*-object questions (*what* questions)
example	target: Was machst du hier? (“What are you doing here?”)	target: Was magst du fressen? (“What do you like to eat?“)
*wh*-adjunct questions	*n* = 6 (*where/when/how* questions)	*n* = 6 (*where/how* questions)
example	Wo wohnt die Oma? (“Where is the grandma living?“)	Wo wohnst du? (“Where are you living?“)
Example of dialogue / instruction (in italics)	Examiner: Die Schnecke spricht leider nicht mit Erwachsenen, nur mit Kindern. *Frag die Schnecke, was sie hier macht.* (“Unfortunately, the snail does not speak to adults. *Ask the snail what it is doing.*”) Child: Was machst du hier? (“What are you doing here?”) Examiner (takes the role of the snail): Ich besuche jemanden. (“I am visiting someone.”)	Examiner: Das Tier ist in der Box versteckt. *Frag das Tier, was es fressen mag.* („The animal is hidden in the box. *Ask the animal what it would like to eat.”*) Child: Was magst du fressen? (“What do you like to eat?“) Examiner (takes the role of the hidden animal): Ich fresse gern Bananen. (“I like to eat bananas.”)

Accuracy scores for *wh*-question production were determined for all utterances that contained an overt *wh*-element and/or displayed a clear raising question intonation. Questions were judged as syntactically correct when there was a fronted *wh*-word, a finite verb in second position, and a subject (which could also be the *wh*-word).

#### 2.2.2. Cognitive measures as potential predictors for grammatical development (RQ 2)

As we were not only interested in the development of the receptive and expressive grammatical abilities but also wanted to evaluate whether nonverbal cognition and verbal short-term memory play a role in determining the development in these areas, the following measures were included to assess these variables.

##### 2.2.2.1. Nonverbal cognition: reasoning scale of the SON-R 2.5–7

The Reasoning Scale of the Snijders-Omen Nonverbal Intelligence Test (SON-R 2.5–7; Tellegen et al., 2007) was used to assess nonverbal cognitive abilities. It consists of three subtests (Categories, Analogies, Situations) that test concrete and abstract reasoning skills. It is normed for the ages of 2;6 to 7;11 years. Reported internal consistency of the Reasoning Scale is *r* = 0.83. Validity is confirmed by a high correlation (*r* = 0.74) with the nonverbal scale of the K-ABC (Melchers and Preuß, 2001; Tellegen et al., 2007). Total raw scores were used in the analyses.^5^ In addition, nonverbal mental age equivalents were computed to describe the sample.

##### 2.2.2.2. Verbal short-term memory: nonword repetition subtest of the SETK 3–5

A common task to assess verbal short-term memory is the repetition of nonwords. It is also well-suited for individuals with Down syndrome and has been used frequently in previous investigations (e.g., Laws and Gunn, 2004; Conners et al., 2018). In the current study, the nonword repetition subtest of the SETK 3–5 (Grimm, 2001) was employed. It comprises 18 nonwords with a length of two to five syllables. The nonwords were read to the participants, who had to repeat them accurately immediately after presentation. Raw scores, i.e., the number of correctly repeated nonwords, were used in the analyses (max. 18 points). Internal consistency reliability ranges between 0.73 and 0.81, depending on the age band (Grimm, 2001). Correlations with other measures of verbal short-term memory to provide information about validity are not reported.

### 2.3. Procedure

Data collection at T1 took place between 2013 and 2015 either in a quiet room at the university or at participants’ homes. A broad range of language and cognitive measures, both experimental and standardized, was administered in four sessions (40–60 min). Here, we report only those measures which were repeated at second testing (T2). Testing at T2 was carried out between 2018 and 2020 and took place at participants’ homes or in institutions for language therapy. A subgroup of five individuals was tested again at T3. Testing at T3 took place in 2022 and only included the measures of expressive syntax. Table 3 presents an overview on which tests were conducted at T1, T2, and T3.

**Table 3:** Number of participants that were tested at T1, T2, and T3.

	Nonverbal cognition	Verbal short-term memory	Receptive grammar	Expressive grammar		
Testing	Reasoning scale of the SON-R	Nonword repetition	TROG-D	Subject-verb agreement	*wh*-questions task 1	*wh*-questions task 2
T1	17	17	17	17	14	–
T2	17	16	17	5	6	7
T3				5	5	–

In all testing sessions sufficient time for pause was given. The order of presentation of the different measures was usually the same, with an alternation of receptive and expressive tasks where possible. Standardized measures were applied according to the procedure described in the manuals. Testing took place after a time of familiarization of the participant with the examiner and the situation. Where possible, parents were not present during the testing sessions to avoid distraction.

Each session was audio- and videorecorded. Recordings were used for the transcription of participants’ verbal responses or to check the scoring of participants’ nonverbal responses in the tests on receptive grammar and nonverbal cognition. Responses in the tasks on subject-verb agreement and *wh*-question production were transcribed by a primary transcriber and transcripts were checked by a secondary transcriber. If necessary, disagreement was resolved with the assistance of a third qualified person. Utterances for which no interrater agreement could be achieved were not included in the analyses.

Approval for data collection was obtained by the Ethics Committee of the Medical Department of the University of Cologne (numbers of approvals: 12-033, 18-121). Informed written consent was given by the parents or legal guardians of all participants and verbal consent from the participants was obtained on the test date.

### 2.4. Analyses

As raw scores are measured on an ordinal scale, non-parametric procedures were chosen.

#### 2.4.1. Analyses addressing RQ1a,b

To find out if there was a significant group change between T1 and T2 in the different measures, Wilcoxon signed rank tests were computed. For the examination of individual change, difference scores for each participant and variable were determined by subtracting the result achieved at T1 from the result achieved at T2. Positive scores show progress, whereas negative scores indicate a decline in performance (see Table 4 for means, standard deviations, and range). To gain more insight into the time course of the development, we determined whether change in language measures was related to the chronological age of the participants. Therefore, we calculated Spearman’s correlations between individual difference scores and chronological age at T1. To gain a more comprehensive picture of the development of expressive grammatical abilities, we performed a post-hoc exploratory analysis of the data for the expressive tasks where we looked for implicational relationships between the tested phenomena.

**Table 4:** Means (standard deviations) and ranges for first (T1) and second testing (T2) as well as difference scores (T2 minus T1).

		Time 1			Time 2		Difference	
	*N* T1	Mean (*SD*)	Range	*N* T2	Mean (*SD*)	Range	Mean (*SD*)	Range
Nonverbal mental age in months	17	56.2 (12.0)	41–77	17 (15)^a^	68.3^a^ (26.1)	44–>96^a^	14.3^a^ (11.4)	(−7)–29^a^
Reasoning scale raw scores	17	24.8 (5.7)	17–33	17	31.0 (6.4)	19–42	6.2 (4.3)	(−2)–12
Nonword repetition raw scores	17	6.1 (3.7)	0–13	16	7 (3.1)	1–11	1.3 (2.6)	(−3)–8
TROG-D raw scores	17	6.1 (2.7)	3–11	17	8.9 (2.8)	4–16	2.8 (2.5)	0–7
Subject-verb agreement accuracy scores	17	65.4% (28.4)	16.7–100%	5	85.3% (18.1)	44.4–100%	19.8 (19.4)	(−6.3)–54.9%
*wh*-production accuracy scores	14	58.61% (36.0)	0–100%	9	81.0% (30.8)	10–100%	25.6% (24.2)	0–80%

a The nonverbal mental age of two participants at T2 could not be determined exactly because their performance exceeded the norming sample of the SON-R 2.5–7. It can therefore only be estimated as at least 8;0 years (96 months). These participants were not included in the calculation of the mean mental age at T2 and the difference scores for mental age.

#### 2.4.2. Analyses addressing RQ2

Analogous to the procedure for RQ1, we first examined group changes in the measures of nonverbal cognition and verbal short-term memory themselves using Wilcoxon signed rank tests. Individual changes were further explored descriptively. To investigate the relation between changes in receptive or expressive morphosyntactic abilities (difference scores) and nonverbal cognition (reasoning scale raw scores) or verbal short-term memory (nonword repetition scores) as possible influencing factors correlational analyses were performed for these measures.

## 3. Results

An overview of the results for T1 and T2 and the difference scores can be found in Table 4. Individual test results achieved at T1, T2, and T3 can be found in the Supplementary material to this text. No overall floor or ceiling effects could be observed in the measures used in this study.

### 3.1. Receptive grammar (RQ1a)

**Figure 1:**
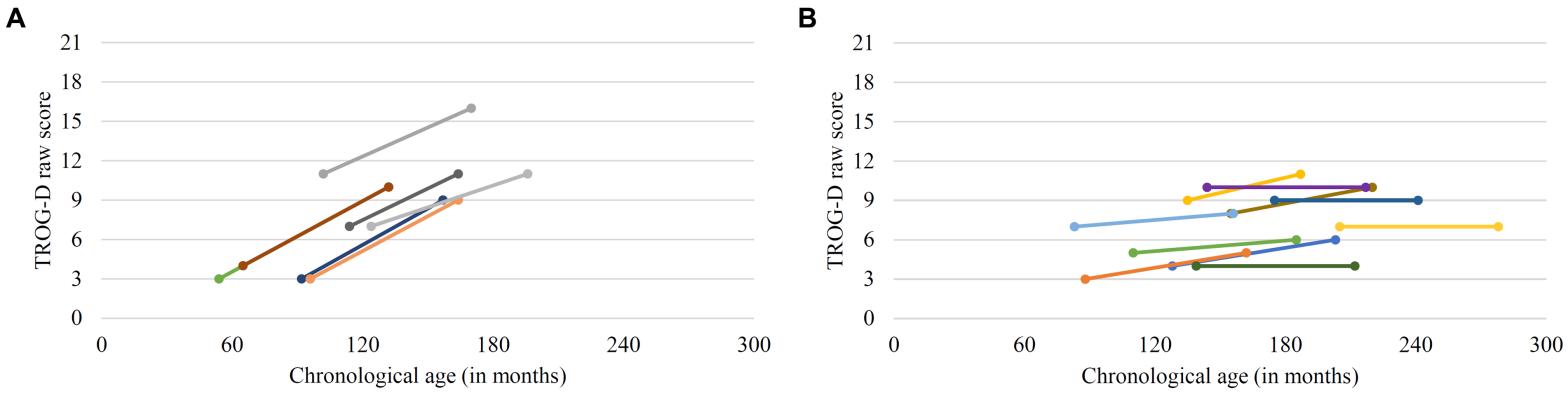
Individual change in Test for Reception of Grammar (TROG-D) raw scores. **(A)** Participants with notable change (difference scores ≥ 3) **(B)** Participants with little or no change.

There was a significant increase in overall TROG-D raw scores between T1 and T2 (*z* = 3.195, *p* = 0.001, *r* = 0.775; *Mdn* T1 = 7, *Mdn* T2 = 9). However, difference scores were negatively correlated with chronological age at T1 [*r_s_*(15) = −0.717, *p* = 0.001], indicating that older participants exhibited less growth in TROG-D scores than younger participants. The individual data obtained at T1 and T2 are presented in Figure 1. To obtain more information on the time course of the development, individual change was further explored descriptively. The data show that a notable increase in raw points only occurred in individuals with a chronological age of 10 years or younger at T1. Since statistical measures to analyze individual change were not available for our data, an increase of three or more raw points was considered as notable. The oldest participant that achieved an improvement of three or more raw points in this test was aged 10;4 years at T1 (see Supplementary material). In contrast, for participants that were older than 10 years at T1, raw scores changed little or not at all (see Figure 1; Supplementary material).

### 3.2. Expressive grammar (RQ1b)

#### 3.2.1. Subject-verb agreement

The majority of the sample (12 out of 17 individuals) already performed well at subject-verb agreement at T1 and achieved accuracy scores of over 85% (range 86.2–100%, mean 94.6%), leaving only five participants with the potential to improve notably by T2. The development of these five individuals was followed up on and is presented here and in Table 4. Also, due to the small number of data points at T2, we did not perform statistical tests. Of the five individuals that displayed problems with subject-verb agreement at T1 (range of accuracy scores 25–54.2%, mean 40.8%) three were younger and two older than 10 years (chronological age). All five individuals showed an improvement in accuracy scores at T2 (mean 70.5%, range 44.4–82.1%). To further investigate the progress of this group of individuals, the group was retested again at T3. However, further progress to accuracy scores of over 90% could only be determined for two of the five tested individuals, both younger than age 10 years at T1 (see data for P1 and P6 in the Supplementary material). For the other three individuals (P4, P13, and P14) no further progress occurred, instead accuracy scores declined from a mean score of 63% at T2 to a mean score of 51.6% at T3 (range of accuracy scores at T3 25–65.5%).

#### 3.2.2. *Wh*-question production

**Figure 2:**
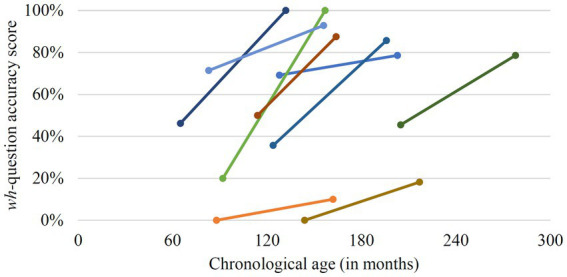
Individual change in *wh*-question production accuracy scores between T1 and T2.

The within-group comparison for *wh-*question production is based on the data of nine participants. Five participants achieved high accuracy scores (over 90%, range 90.9–100%) already at T1 and, therefore, displayed only a limited potential for improvement at T2. For three other individuals, it was not possible to perform the question elicitation task at T1 due to lack of cooperation, insufficient understanding of the task, and/or insufficient language skills. Thus, a difference in accuracy scores to T2 could not be determined. The comparison of T1 and T2 performance for the nine participants indicates significant growth between the two assessments in the group as a whole (*z* = 2.666, *p* = 0.008, *r* = 0.889; *Mdn* T1 = 45.45%, *Mdn* T2 = 85.71%). The mean accuracy score of 37.6% at T1 (range 0–71.4%) increased to 72.5% at T2 (range 10–100%). An inspection of the individual data reveals that seven of the nine individuals achieved a considerable improvement in accuracy scores for *wh*-question production between T1 and T2 (see Figure 2). For two individuals (P4 and P14), however, accuracy scores still were below 20% at T2, indicating a clear deficit in expressive grammatical abilities that persisted at T2. The correlation between *wh*-question production difference scores and chronological age at T1 did not yield a significant result [*r*_s_(7) = −0.317, *p* = 0.406]. Thus, for the tested nine children and adolescents, the improvement in *wh*-question production seemed to be independent of their chronological age.

To determine the development in producing *wh*-questions in the three individuals who could not perform the task at T1 (P1, P6, and P13) and the two individuals who failed to produce *wh*-questions at T1 and only achieved minimal improvement at T2 (P4, P14), we retested *wh*-question production for these participants at T3. Two of the participants, who could not perform the task at T1 (P1 and P6), achieved accuracy scores of 38% and 58% at T3, indicative of an improvement in producing syntactically correct *wh*-questions. For the other three individuals (P4, P13, and P14), no substantial increase in accuracy scores could be observed at T3, and accuracy scores remained very low (range 0–18%).

#### 3.2.3. Exploratory analysis for expressive grammar

With regard to their expressive morphosyntactic abilities the participants fall into three different groups. The first group, consisting of five individuals (P7, P8, P12, P15, and P16), achieved accuracy scores of over 80% in both expressive measures, indicating good performance with subject-verb agreement marking *and* the production of *wh*-questions already at T1.

The second group of seven individuals (P2, P3, P5, P9, P10, P11, and P17) had already obtained high accuracy scores of over 80% for subject-verb agreement at T1 while accuracy scores for *wh*-question production were lower at T1 and only reached 80% or more at T2, indicating that the development of *wh*-question production proceeded after subject-verb agreement marking had been mastered.

The third group of five individuals (P1, P4, P6, P13, and P14) obtained relatively low scores in both expressive grammatical measures at T1 (range of accuracy scores for subject-verb agreement at T1 25–54.2%, accuracy scores in *wh*-question production at T1 0% if test could be performed). At T2 and T3, two of these individuals (P1 and P6) achieved accuracy scores of over 80% for subject-verb agreement while the accuracy scores for *wh*-question production did not reach this level at T3 (58.3 and 38.5%). For the other three individuals, accuracy scores for verbal agreement marking surpassed the accuracy scores for *wh*-question production at T2 and T3. However, none of these individuals achieved an accuracy score of 80% or above for either of the two expressive morphosyntactic measures at T3. For all three testing times, the data, thus, yield that progress in subject-verb agreement marking precedes progress in the production of *wh*-questions. The data do not contain a single case where an individual achieved good performance in the production of *wh*-questions but was impaired in the marking of subject-verb agreement.

This order of difficulty between performance in subject-verb agreement and production of *wh*-questions was confirmed by an implicational scaling analysis of the accuracy scores obtained by the participants for subject-verb agreement and for *wh*-question production at T1 (Guttman, 1944; Hatch and Lazaraton, 1991). The analysis was conducted to determine whether the acquisition of subject-verb agreement and the acquisition of *wh*-questions (both defined by an accuracy score of over 80%) display a scale, indicating that acquisition of the one phenomenon truly precedes acquisition of the other. As the data do not contain a single case where participants display better performance with respect to the production of *wh*-questions compared to the production of subject-verb agreement marking, the implicational analysis gave a perfect *Guttman coefficient of scalability* (= 1). The coefficient allows to predict with 100% accuracy that an individual displaying good performance (> 80% accuracy) with respect to the production of *wh*-questions will also achieve good performance with the marking of subject-verb agreement. The analysis, thus, implies a true developmental scale according to which acquisition of subject-verb agreement marking precedes the production of *wh*-questions.

### 3.3. Potential predictors for grammatical development (RQ2)

#### 3.3.1. Nonverbal cognition

There was a significant increase in reasoning scale raw scores in the overall group (*z* = 3.364, *p* < 0.001, *r* = 0.816; *Mdn* T1 = 24, *Mdn* T2 = 31). On an individual level, raw scores increased for all participants except for two, who showed a decrease of 2 raw points or no change. Difference scores for the others ranged between 1 and 12. There was no significant correlation of reasoning scale difference scores with chronological age [*r*_s_(15) = −0.417, *p* = 0.096], indicating that the improvement in nonverbal cognition was independent of participants’ chronological age at T1.

#### 3.3.2. Verbal short-term memory

**Figure 3:**
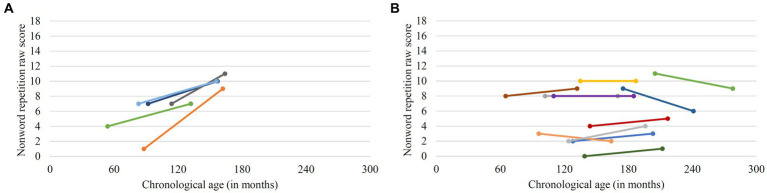
Individual change in nonword repetition scores. **(A)** Participants with notable change (difference scores ≥ 3) **(B)** Participants with little or no change or decline.

There was no significant growth in nonword repetition scores in the group as a whole between T1 and T2 (*z* = 1.870, *p* = 0.062, *r* = 0.468; *Mdn* T1 = 7, *Mdn* T2 = 8). Note that one participant was missing a score at T2 as the test could not be performed due to lack of cooperation. Nonword repetition difference scores correlated negatively with chronological age at T1 [*r*_s_(14) = −0.566, *p* = 0.022]. This indicates that older participants at T1 displayed less growth or even a decline in nonword repetition performance compared to younger participants. Figure 3A shows that participants with a notable change in nonword repetition scores (here defined as an increase in raw scores of three or more raw points) were younger than 10 years at T1 (chronological age). In contrast, participants that were older than 10 years at T1 displayed little or no change in raw scores for nonword repetition (Figure 3B). The oldest participant that achieved an improvement of three or more points in this task was 9;6 years at T1.

#### 3.3.3. Relation of language change and nonverbal cognition or verbal short-term memory

The results of the correlational analyses that were performed to investigate the relation between language difference scores and performance on the reasoning scale of the SON-R or nonword repetition performance at T1 are displayed in Table 5. Chronological age was controlled in the correlations with reasoning scale raw scores as both were positively related [*r_s_*(15) = 0.644, *p* = 0.005]. Analyses yielded that the difference scores obtained for receptive grammar (TROG-D difference scores) correlated neither with reasoning scale raw scores, measuring nonverbal cognition, nor with nonword repetition performance, our measure for verbal short-term memory capacities.

**Table 5:** Correlations between chronological age, reasoning scale raw scores, and nonword repetition scores at T1 and TROG-D and *wh-*question difference scores.

	Difference scores	
	TROG-D	*wh*-questions
Chronological age at T1	*r_s_* = −0.717	*r_s_* = −0.317
	*p* = 0.001	*p* = 0.406
	*n* = 17	*n* = 9
Reasoning scale raw scores at T1	*r_s_* = −0.146	*r_s_* = 0.313
	*p* = 0.589	*p* = 0.451
	*n* = 17	*n* = 9
Nonword repetition scores at T1	*r_s_* = −0.122	*r_s_* = 0.545
	*p* = 0.640	*p* = 0.129
	*n* = 17	*n* = 9

Correlations with reasoning scale raw scores were controlled for chronological age.

For expressive grammar, correlational analyses were only performed for the accuracy difference scores obtained for *wh*-question production from those nine individuals that were tested at T1 and T2. *Wh-*question production difference scores did not correlate with reasoning scale raw scores or nonword repetition scores at T1. For subject-verb agreement, no correlations between accuracy difference scores and SON-R reasoning scale or nonword repetition were computed, since the number of data points at T2 was too small (only five individuals). Note however, that the three participants who displayed no progress with respect to verbal agreement marking (P4, P13, and P14) nevertheless progressed with respect to their nonverbal cognitive abilities (increases in raw scores of 4, 8, and 9 points). This suggests that progress in nonverbal cognitive development is not linked to progress in the production of verbal agreement markings.

## 4. Discussion

### 4.1. Development of receptive and expressive morphosyntactic abilities (RQ1)

The main purpose of our study was to investigate the developmental course in receptive and expressive morphosyntactic abilities in individuals with Down syndrome. To this end, we analyzed data from 17 individuals with Down syndrome that were collected at two time points about 4½–6½ years apart. For a subset of five participants, there was also an additional third assessment 2 years after the second.

Regarding grammar comprehension, we found a significant improvement between T1 and T2 in the group as a whole. However, this did not apply to all participants. A negative correlation of TROG-D difference scores with chronological age at T1 showed that there was less improvement in older individuals, suggesting a levelling off in the development of receptive grammatical abilities. Closer inspection of the individual data indicated that this occurs around the age of 10 years. This finding is in accordance with other investigations that have also reported diminished or ceasing progress in this domain around the age of 10–12 years (Chapman et al., 2002; Laws and Gunn, 2004; Conners et al., 2018). In contrast to the results by Chapman et al. (2002) and Laws and Gunn (2004), we did not find declining performance in grammar comprehension in our sample. However, in these studies decline was mostly observable in late adolescence and early adulthood, setting off around the age of 17 years. As the participants in the present study were younger than 17 years at study start (one exception) it remains open if they will possibly be affected by decline when they get older.

In the expressive domain, a large part of the investigated sample (*n* = 12) already performed well in verbal agreement marking at T1 and therefore only had limited potential for further development in this area. The descriptive analysis of the remaining five participants’ results at T2 and T3 revealed consistent improvement only for two individuals. Both achieved a good proficiency with verbal agreement marking (accuracy score of over 80%) at some point between T1, when they were younger than 10 years, and T2 when they were slightly older than 10 years (chronological age: 11 and 13 years). In contrast, for three participants no consistent progress could be determined. They had not succeeded in mastering the system of verbal agreement marking by T3 when they were 15–20 years old. Thus, taking into account the small number of participants one can cautiously conclude that individuals who have not reached a high level of proficiency with respect to subject-verb agreement marking by late childhood might not display further development in this grammatical domain and, hence, do not acquire the German system of subject-verb agreement marking.

For the production of *wh*-questions, we saw high performance at T1 in five participants. The others exhibited a significant positive change when T1 and T2 performance were compared at group level. Difference scores were not related to the chronological age of the participants, suggesting that, in contrast to the receptive domain, improvements in *wh*-question production occurred irrespective of age. However, a closer inspection of the present data indicates that a notable improvement in the production of *wh*-questions was only observable for those participants who had acquired verbal agreement marking. Reversely, little change could be seen in those participants that had not acquired verbal agreement marking until late childhood (i.e., around the age of 10 years). The data, thus, suggest an implicational relationship between the acquisition of the verbal agreement system and progress in the production of *wh*-questions: progress in *wh*-question production could only be observed in those individuals who had mastered the system of verbal agreement marking.

The implicational relationship between these two phenomena is reminiscent of morphosyntactic development in typically-developing two-to-four-year old German-speaking children where the mastery of the verbal agreement system also precedes the production of *wh*-questions (Clahsen and Penke, 1992; Penke, 2001). The developmental relation between verbal agreement inflection and the production of *wh*-questions is rooted in the V2-property of German: in main clauses the finite verb, i.e., the verb that is marked for subject-verb agreement, moves to the second structural position in the clause. In the framework of generative syntax, this is achieved by movement of the verb from a position within the verbal phrase (VP), which encodes the argument structure of the described event, to functional projections which serve syntactic functions. An inflectional phrase (IP) takes care of the agreement inflection between subject and verb; another functional phrase (CP) accounts for the appearance of the finite verb in the second structural position in the sentence. To appear in this syntactic position the verb undergoes two syntactic movement operations: It first moves from the head of the VP to the head of the IP to enter into an agreement relation with the subject. Subsequently, it moves from the head of the IP to the head of the CP to appear in the second structural position (Haegeman, 2001).

**Figure 4:**
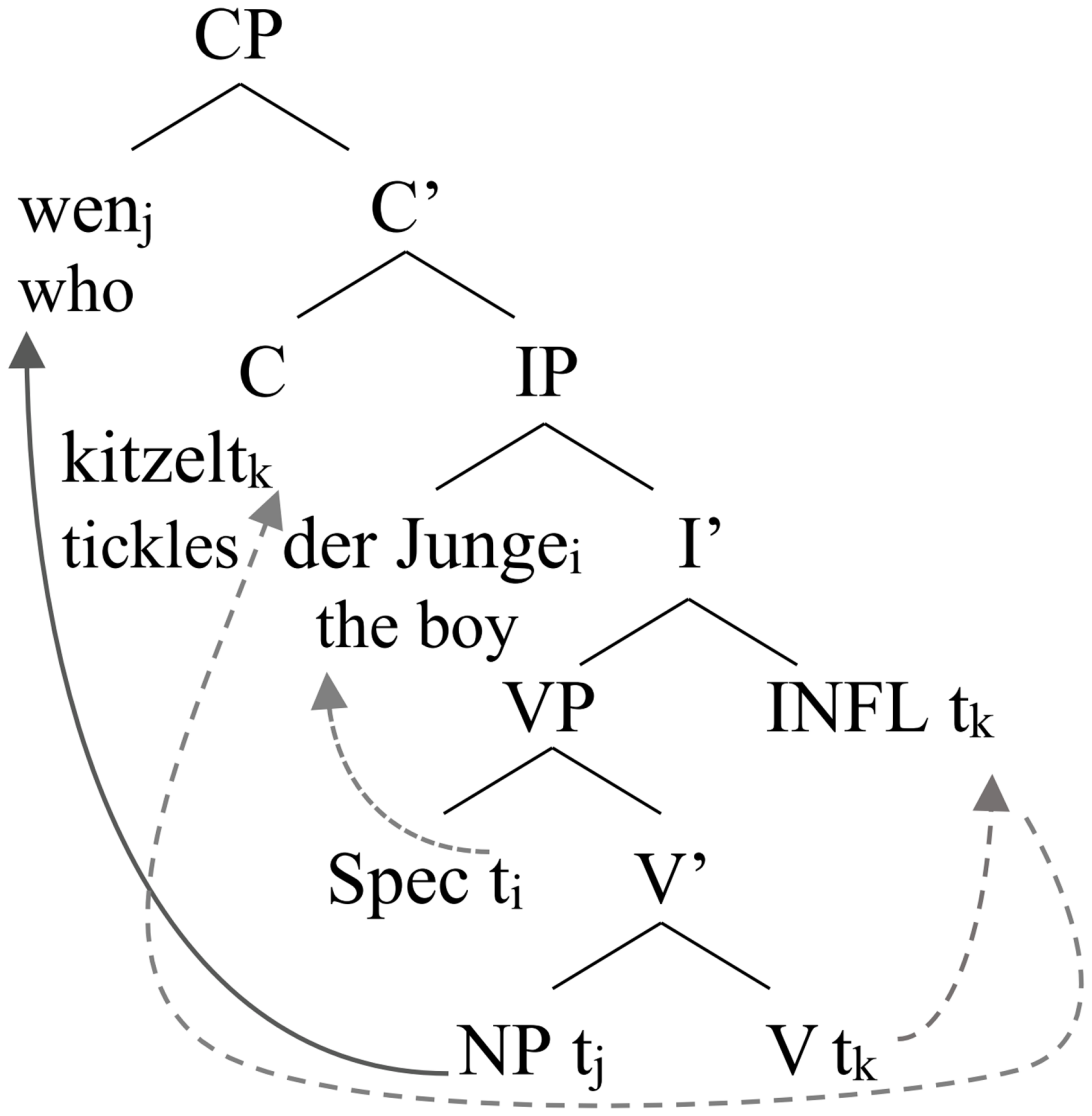
Syntactic tree of the *wh*-question *Wen kitzelt der Junge?* (“Who is the boy tickling?”).

An exemplification is presented in Figure 4 which displays the syntactic tree associated with a short German *wh*-question such as *Wen kitzelt der Junge?* (“Who is the boy tickling?”). In the VP, the verb describing the action (*kitzeln* “tickle”) occupies the head position, the Agent of the action (“the boy”) is situated in the specifier position of the VP (SpecVP) and the Theme/Patient of the action is lexicalized by a *wh*-pronoun in the complement position of the verbal head. In a first round of syntactic operations, the Agent moves to the specifier position of the IP where it is marked as subject by receiving nominative case inflection. The verb moves to the head of the IP to enter into an agreement relation with the person and number specifications of the subject, expressed by subject-verb agreement markers on the verb. Each moved constituent leaves behind an indexed trace (*t*) that connects the moved constituent to its base position in the VP. In the next round of syntactic operations, the finite verb moves from the head of the IP to the head of the CP position to occupy the second structural position in the sentence. V2 word order then comes about by movement of another sentence constituent, here the *wh*-pronoun, to the specifier position of the CP, the sentence initial position (so-called *wh-movement*).

In the lexical learning framework proposed by Clahsen et al. (1996), the acquisition of the verbal agreement system that takes place between the ages of two-to-three in typically-developing German-speaking children brings about the build-up of both functional phrases, the IP and the CP in the syntactic tree, thus, enabling the V2 movement of finite German verbs. The acquisition of the verbal agreement system leads to the build-up of the IP in the syntactic tree. With the acquisition of subject-verb agreement, the child can now identify which verbs move to the second structural position in main clauses, the head of CP, i.e. verbs inflected for subject-verb agreement. Moreover, s/he has acquired the means to inflect all verbs for agreement with the subject. Besides the head position for the finite verb, the CP contains a specifier position (SpecCP) that can now be filled with a *wh*-phrase moved out of its position in the VP. Movement of the *wh*-phrase to sentence-initial position can, thus, only occur after the build-up of the CP layer (by age 3 to 4 years in typically-developing German-speaking children) that is itself connected to the acquisition of subject-verb agreement marking. This is in line with the results of the implicational analysis reported above which showed that mastery of the verbal agreement system consistently preceded the ability to produce syntactically correct *wh-*questions in our participants. This observation suggests that the acquisition of the system of verbal agreement marking might have a trigger function for further grammatical development in individuals with Down syndrome. Moreover, our data suggest that the building of the syntactic tree needs to be completed within a certain time window, around the age of 10 years (chronological age), to enable further grammatical development with respect to syntactic structures that require the projection of the CP layer, such as *wh-*questions. While this is an intriguing suggestion, its data base is small and requires further investigation.

Note that while the acquisition of the system of subject-verb agreement inflection plays a central role for the acquisition of syntactic structures in German, this does not hold across languages. The lexical learning approach to syntactic development assumes that the acquisition of functional heads proceeds when children acquire the bound or free grammatical morphemes that lexicalize these functional heads in the language they acquire (Clahsen et al., 1996). While this acquisition procedure holds across languages, the lexical elements that lead to the build-up of functional phrases are language-specific. Thus, while the implicational relationship between the acquisition of subject-verb-agreement and the build-up of the CP layer holds for German, future research would have to target whether similar implicational relationships characterize the acquisition of syntactic structures in other languages and whether developmental restrictions in the timely build-up of the syntactic tree can also be observed in individuals with Down syndrome that speak other languages than German.

Our suggestion that there is a critical window for the acquisition of verbal agreement inflection and, concomitant, the building of the syntactic tree, and that only if this is accomplished, further grammatical development might come about in German-speaking individuals with Down syndrome conforms to the findings of Conners et al. (2018). In their longitudinal study, they observed no progress with respect to expressive grammatical abilities in the tested individuals with Down syndrome. Interestingly, their participants were aged 10 to 21 years at T1, suggesting that a critical time window for further syntactic development (around the chronological age of 10 years) might already have been closed for their participants. This supports our findings and the suggestion that there is a critical window for the development of expressive grammatical abilities in individuals with Down syndrome that might close around late childhood.

Taken together, our data indicate a critical time window for the development of receptive as well as expressive grammatical abilities in individuals with Down syndrome that seems to close around late childhood. Within this critical time window there is the possibility of further progress in receptive and expressive grammatical abilities. However, this does not imply that progress within this critical time window is guaranteed to occur. In the present study, for instance, there was one participant (P4) who displayed no consistent progress in receptive and expressive grammar measures despite an age of only 7 years at T1. More research is needed to confirm the conclusions drawn from the present data and to determine the factors that advance or hinder further development of grammatical abilities within the critical time window in individuals with Down syndrome.

### 4.2. Relation between morphosyntactic development and nonverbal cognition as well as verbal short-term memory (RQ2)

A further goal of our study was to investigate whether individual grammatical development is influenced by nonverbal cognitive abilities or verbal short-term memory capacity, two factors that have been discussed as predictors of grammatical development in the literature.

With respect to progress in nonverbal cognition, we found ongoing development in most of the examined individuals irrespective of their chronological age. The finding of continuing growth of nonverbal cognitive abilities in adolescence in individuals with Down syndrome is consistent with existing studies (Couzens et al., 2011; Channell et al., 2014). It contrasts, however, with the stagnation of receptive and expressive grammatical abilities that we observed in participants after late childhood. Correlational analyses between reasoning scale raw scores at T1 and difference scores for our grammar measures did, therefore, not reveal a relation. Specifically, it was not the case that participants with higher scores in our measure of nonverbal cognition at study start showed more improvement in either receptive or expressive grammatical abilities than those with lower scores, a relation that one might have expected given the findings of previous cross-sectional research that found positive correlations between nonverbal cognitive and language performance (Chapman et al., 1991; Abbeduto et al., 2003; Aktaş, 2004; Price et al., 2007, 2008; Iacono et al., 2010; Estigarribia et al., 2012; Finestack et al., 2013). Our finding that progress in nonverbal cognition is not related to progress in grammar development is, however, in accordance with Chapman et al. (2002) who also found that their measure of nonverbal cognition did not add to the predictive power in their models for syntax comprehension and production. Although our data base is limited, especially with respect to the relation between expressive grammatical abilities and nonverbal cognition, the data presented here and the data of the other longitudinal study investigating nonverbal cognitive and language development (i.e., Chapman et al., 2002) provide converging evidence that development in nonverbal cognitive abilities in children and adolescents with Down syndrome does not proceed hand in hand with ongoing development in grammatical abilities. This does not preclude the possibility that such a relation might hold for very young children with Down syndrome, an issue that should be targeted by further research.

With respect to the development of verbal short-term memory capacity, measured via nonword repetition, the present study indicated that growth in this domain also levelled off early (around the chronological age of 10 years). There was no significant difference in nonword repetition performance between T1 and T2 at group level. Moreover, performance in nonword repetition displayed a negative correlation with chronological age, indicating less growth or even a decline in nonword repetition performance in older compared to younger participants. Notable individual improvement did not occur after the age of 10 years and decline was observable in three individuals, two of them being the two oldest. An early termination in the development of verbal short-term memory capacity has also been reported by Conners et al. (2018) and Laws and Gunn (2004). In their studies, nonword repetition performance even declined in most participants with a chronological age over 10 years. Concerning the role of verbal short-term memory for grammatical development, the correlational analyses provided no indication that participants with better performance in the verbal short-term memory task at study onset exhibited larger growth in receptive or expressive grammatical abilities. The finding for the expressive domain conforms to the analyses by Chapman et al. (2002), where verbal short-term memory also did not prove to be a predictor for growth in grammar production. With respect to receptive grammar, a significant relationship to verbal short-term memory as found in other longitudinal studies (Chapman et al., 2002; Laws and Gunn, 2004) is not confirmed by the present results. Note, however, that the correlation between T1 nonword repetition scores and T2 performance in receptive grammar in Laws and Gunn’s (2004) investigation was only evident in a subsample, aged below 14;8 years at T2. This younger subsample also showed more consistent progress in grammar comprehension than the older participants. Taken together, there is no indication that verbal short-term memory performance is the decisive factor for grammatical development in individuals with Down syndrome.

### 4.3. Other potential predictive factors

Apart from chronological age, nonverbal cognitive abilities or verbal short-term memory capacity, there are others factors that could potentially be related to language progress in individuals with Down syndrome. One factor, that comes to mind, is ongoing support in the form of speech and language therapy. However, a subsequent inspection of this aspect in our participants did not reveal a relation between improvements in language abilities or the lack thereof and the application of language intervention. Of the ten participants that showed little or no change in receptive grammatical abilities between T1 and T2 five had received speech and language therapy during the entire time between T1 and T2. The other half did not obtain language intervention or, in one case, only for a limited part of the time. Furthermore, all five individuals that displayed ongoing difficulties with the production of *wh*-questions received language intervention throughout the duration of the study. Thus, it rather seems to be the case that more severe limitations in language abilities, especially in the expressive domain, are met with prolonged therapeutic services.

Another factor that might be beneficial for language development is the acquisition of literacy. Information on the participants’ reading abilities was only collected at T2. Therefore, it cannot be determined whether the degree of literacy had any influence on the grammatical development of the investigated individuals between T1 and T2. However, at T2 only one participant could not read and one could only read short, frequently occurring words. Three participants had reading skills at sentence level. The majority of the sample (12 out of 17) was able to read at text level. That included participants who did not show substantial progress in receptive or expressive grammatical abilities which suggests that for them literacy did not advance grammatical development.

In addition, we did not find a relation between parents’ level of education, measured on a 9 level scale (ranging from 0 = early childhood education to 8 = doctoral degree) according to the International Standard Classification of Education (United Nations Educational, Scientific and Cultural Organization, 2012), and language change (Spearman’s correlations between mother’s and father’s level of education and difference scores for receptive grammar and *wh*-production: *p >* 0.5 each).

To summarize, the present data, even though limited, do not provide evidence that factors other than chronological age, such as speech and language therapy, literacy or parents’ level of education, determine which participants show progress and which exhibit little or no improvement.

### 4.4. Limitations

There are some limitations of the current study we would like to address. The first issue concerns the relatively small sample size of 17 individuals with Down syndrome—and an even smaller sample in the analyses of expressive grammar—in our study that limits statistical analyses. Although, we started with a relatively large sample size of 31 participants at T1, a large number of these could unfortunately not be recruited again for subsequent testing at T2. In addition, the age range was quite large. Furthermore, the time intervals between T1 and T2 varied between 4½ and 6½ years. Despite these limitations, core findings of our results—such as the levelling off in the development of receptive grammar—concur with previous studies that tested larger samples (e.g., Conners et al., 2018). Thus, a consistent picture of the development of expressive and receptive grammatical skills emerges, pointing to a critical developmental window of about 10 years of age. In future studies, however, more testing points with equal time intervals before and after the age of 10 years should be scheduled, to determine the time window for the acquisition of specific grammatical skills in individuals with Down syndrome more exactly.

Another issue concerns the composition of the sample. Due to the inclusion criteria, such as monolingualism and verbal means of communication, the sample might not be truly representative of the population of individuals with Down syndrome. Furthermore, other background data such as information on ethnicity or adaptive functioning was not available and should be gathered in future studies. Likewise, more detailed information on the methods, goals, and intervals of past therapeutic interventions would be desirable to explore which factors limit or boost an individual’s potential for grammatical development.

Regarding experimental procedures, contrary to other studies (e.g., Chapman et al., 2002), we did not use MLU as a global measure for expressive grammar but focused on specific morphosyntactic phenomena. This limited the comparability of our results to previous findings regarding the development of expressive grammatical abilities. Also, given that only a limited set of morphosyntactic phenomena can be tested without overtaxing the participants, an advance selection had to be made. Thus, we might have missed aspects of morphosyntax that exhibit different developmental patterns in individuals with Down syndrome. Another limitation is the use of two different, albeit very similar, tasks to assess the production of *wh-*questions at T2. Also, information on reliability and validity is not available for the experimental tasks that were used to test expressive grammar.

The SON-R 2.5-7 was used to assess nonverbal cognition. The limited age band of the SON-R norming sample did not allow to calculate IQ scores for most participants to provide them as background information. Therefore, we reported nonverbal mental age equivalents to describe the sample. A ceiling effect in this regard was evident for two participants at T2. Thus, only their minimum mental age could be determined. Note, that nonverbal mental age equivalents have several limitations (see Maloney and Larrivee, 2007 for a comprehensive examination of age equivalent scores).

Ability scores such as growth scale values, that are weighted for item difficulty and measured on an interval scale, are not available for the standardized tests that were used in this study. Hence, we relied on raw scores which do not follow an equal-interval scale. Statistical measures that would indicate significant individual changes were not applicable to our data. Therefore, we—somewhat arbitrarily—considered an increase of three or more raw points as a notable increase in TROG-D or nonword repetition performance. However, as indicated by the data (in the figures and the supplementary table), a different setting for this value (to 2, 4, or even more points) would not change our main finding that larger increases only occur in younger participants.

### 4.5. Clinical implications

The results of the present study not only contribute to the understanding of possible developmental patterns in grammar development, but also lead to clinical implications. The focus of speech and language therapy in individuals with Down syndrome should always be based on the respective individual strengths and weaknesses, as identified by comprehensive diagnostic assessments. Nevertheless, some general conclusions can be drawn from the current findings.

The indication of a critical time window for the development of both receptive and expressive grammatical abilities, which might end around the age of 10 years, suggests that this period is particularly important for intervention in the area of grammar. This is highly relevant because, in our experience, supporting improvement in vocabulary or pronunciation is often prioritized over grammar in therapeutic settings, especially in childhood.

Previous research has shown various impairments in language comprehension in individuals with Down syndrome [*cf.* review by Andreou and Chartomatsidou, 2020]. We would therefore like to stress the importance of addressing not only expressive but also receptive abilities in language therapy, especially since limitations in language comprehension can easily be overlooked or remain unrecognized without suitable diagnostic instruments. Given the concurrent findings of a developmental window closing in late childhood, it is necessary to sensitize educational and therapeutic professionals for receptive deficits and the need for early detailed assessment and intervention in this respect. Therapeutic work on receptive grammar might also have a positive impact on grammar production. For example, Chapman et al. (2000) identified receptive language skills of people with Down syndrome as a crucial predictor for their productive abilities (MLU and number of different words in narration). Future intervention studies should therefore evaluate whether transfer effects from the receptive to the expressive language domain are indeed possible.

Regarding expressive grammatical development, based on the findings of our study, subject-verb agreement can be hypothesized to be a critical structure for the development of the syntactic tree and thus, for further expressive syntactic development in German. If this is indeed the case and if there is a critical time window for the acquisition of verbal agreement, a stronger emphasis on supporting its acquisition within that timeframe could possibly pave the way for further grammatical development in German-speaking individuals with Down syndrome – an issue that probably deserves longitudinal research. There are several therapeutic approaches to support the acquisition of verbal agreement inflection in German (e.g., training of final consonants (TraFiK); Penke et al., 2020; the psycholinguistic approach (PLAN); Siegmüller and Kauschke, 2006). Their effectiveness for individuals with Down syndrome should be investigated in future evaluation studies. Furthermore, to make the best possible use of the described time window, it might be advisable to administer not only regular outpatient speech and language therapy, but also treatment in the form of intensive therapy.

The emphasis we put on targeting grammar in language intervention in early ages to use important time windows for development does, however, not imply that progress in receptive or expressive grammatical abilities of individuals with Down syndrome is impossible beyond the age of 10 years or that therapy should be interrupted or terminated at this age. Progress in expressive grammar seems possible as soon as the syntactic tree is complete. Moreover, targeted speech and language therapy might in fact be necessary to avert stagnation or decline in language abilities, the more so since school support ends after the transition from adolescence to adulthood.

## 5. Conclusion

The findings of the present study indicate that development in receptive grammar levels off in late childhood, around the age of 10 years, confirming previous research. For expressive grammar, mastery of the verbal agreement system consistently preceded the ability to produce syntactically correct *wh-*questions in our participants. Notable improvement in *wh-*question production only occurred in individuals with good performance in subject-verb agreement marking. Thus, our study not only confirmed previous results but expanded them by putting forward the suggestion of a trigger function of the acquisition of the verbal agreement system for further expressive grammatical development in German-speaking individuals with Down syndrome. We hypothesized that the building of the syntactic tree, that is connected to the acquisition of the verbal agreement paradigm, needs to be completed within a certain time window, around the age of 10 years, to enable the acquisition of sentence structures that involve the CP layer, e.g., *wh-*questions. These ideas provide an avenue for future research and should be pursued in studies with larger longitudinal samples and different measures for expressive morphosyntactic abilities.

Our finding of a critical time window for further morphosyntactic development in individuals with Down syndrome has implications for speech and language intervention. Whether targeted intervention in adolescence can help to delay or even prevent the levelling off in grammatical development observed in individuals with Down syndrome, is an important issue to address in future research.

## Data availability statement

The datasets presented in this article are not readily available because they include data from vulnerable participants and third-party availability of the data was not part of the ethics approval. Requests to access the datasets should be directed to BW: bwitecy@uni-koeln.de.

## Ethics statement

The studies involving human participants were reviewed and approved by the Ethics Committee of the Medical Department of the University of Cologne. Written informed consent to participate in this study was provided by the participants’ legal guardian/next of kin.

## Author contributions

MP conceptualized and supervised the study. BW, EW, and IN collected the data. BW and MP analyzed the data. BW wrote the manuscript. EW, IN, and MP contributed to the writing of the manuscript. BW, EW, IN, and MP edited the manuscript. All authors contributed to the article and approved the submitted version.

## Funding

T1 assessments were supported by the German Science Foundation (DFG, grant numbers: PE 683/3-1 and WI 4130/2-1). We acknowledge support for the Article Processing Fee from the DFG (German Research Foundation, 491454339).

## Conflict of interest

The authors declare that the research was conducted in the absence of any commercial or financial relationships that could be construed as a potential conflict of interest.

## Publisher’s note

All claims expressed in this article are solely those of the authors and do not necessarily represent those of their affiliated organizations, or those of the publisher, the editors and the reviewers. Any product that may be evaluated in this article, or claim that may be made by its manufacturer, is not guaranteed or endorsed by the publisher.

## Footnotes

^1^Note that data on grammar comprehension as well as predictor variables has been previously published in a German paper for a German-speaking readership of speech and language therapists (Witecy et al., 2021).

^2^The participants were part of a larger sample that was first examined in a cross-sectional study on the grammatical abilities of children and adolescents with Down syndrome. The initial cohort included 31 children and adolescents. While the initial assessment of this cohort was funded by the German Science Foundation, the follow-up assessments reported in this paper could only take place as personnel, financial and time resources permitted. This accounts for the protracted assessment period at T2. Furthermore, data collection was delayed because of COVID-19 related regulations. Participants of the initial cohort, respectively their parents, were contacted for assessment at T2. However, only 20 families agreed to further participation of which one family had moved too far away and two participants could not be assessed due to increased COVID-19 related restrictions. This resulted in a sample of 17 participants for the follow-up.

^3^For a more detailed description of the task and cross-sectional results for a larger sample, which includes the participants in the current study, see Penke (2018).

^4^Further information on the task and detailed analyses of *wh-*question production in individuals with Down syndrome can be found in Wimmer et al. (2020).

^5^Performing the analyses with nonverbal mental age does not change the results.
