# Supplementary material for: Morphosyntactic development in German-speaking individuals with Down syndrome—longitudinal data
Source: Front Psychol. 2023 Jun 21;14:1118659. doi: 10.3389/fpsyg.2023.1118659 (PMC10321659; doi:10.3389/fpsyg.2023.1118659)
Supplement: Supplementary file 1 [file Data_Sheet_1.PDF]

frontiers

Supplementary Data Sheet. Overview on longitudinal data obtained for individual participants

			nonverbal cognition		verbal short-term memory	receptive grammar	expressive grammar	
Participant Gender	Testing	c.a. y;m	SON-R m.a. y;m	SON-R raw score	nonword repetition (max. 18 points)	TROG-D (max. 21 points)	subject-verb agreement (accuracy score)	*wh*-question production (accuracy score)
P1$_f$	T1	4;6	4;5	24	4	3	42.9%	not possible
	T2	11;0	6;10	36	7	10	82.1%	not tested
	T3	13;0					92.9%	58.3%
P2$_f$	T1	5;5	3;8	19	8	4	96%	46.2%
	T2	11;0	5;5	29	9	10	-	100%
P3$_m$	T1	6;11	4;8	25	7	7	90%	71.4%
	T2	13;0	6;0	31	10	8	-	100%
P4$_m$	T1	7;4	3;8	19	1	3	41.7%	0%
	T2	13;6	4;11	26	9	5	70.4%	10%
	T3	15;5					64.3%	18%
P5$_m$	T1	7;8	3;9	19	7	3	100%	20%
	T2	13;1	5;9	31	10	9	-	100%
P6$_m$	T1	8;0	3;8	19	3	3	40%	not possible
	T2	13;8	5;3	28	2	9	81.3%	not tested
	T3	15;7					96.4%	38.5%
P7$_f$	T1	8;6	4;2	22	8	11	96.7%	91.7%
	T2	14;2	6;3	32	8	16	-	-
P8$_m$	T1	9;2	3;9	19	8	5	93.1%	100%
	T2	15;5	3;8	19	8	6	-	-
P9$_f$	T1	9;6	6;0	31	7	7	96.6%	50%
	T2	13;8	5;5	29	11	11	-	88.9%
P10$_m$	T1	10;4	4;5	24	2	7	96.6%	35.7%
	T2	16;4	5;4	25	4	11	-	85.7%
P11$_m$	T1	10;8	3;5	17	2	4	100%	69.2%
	T2	16;11	3;10	20	3	6	-	78.6%
P12$_m$	T1	11;3	6;2	33	10	9	96.6%	100%
	T2	15;7	>7;11	42	10	11	-	-
P13$_f$	T1	11;7	5;1	27	0	4	25%	not possible
	T2	17;8	6;0	31	1	4	44.4%	not tested
	T3	19;6					25%	0%
P14$_m$	T1	12;0	5;10	33	4	10	54.2%	0%
	T2	18;1	>7;11	41	5	10	74.1%	18.2%
	T3	20;0					65.5%	8.3%
P15$_f$	T1	12;11	5;0	27	13	8	86.2%	90.9%
	T2	18;4	6;10	34		10	-	-
P16$_f$	T1	14;7	5;6	30	9	9	96.6%	100%
	T2	20;1	7;9	38	6	9	-	-
P17$_m$	T1	17;1	6;5	33	11	7	86.2%	45.5%
	T2	23;2	6;10	34	9	7	-	78.6%

Participants ordered according to chronological age (ascending) at T1
Subscript after participant number: f: female, m: male
chronological age (c.a.) and nonverbal mental age (m.a.) given in year;months
/: not tested at T3
